# Delay Minimization for BAC-NOMA Offloading in UAV Networks

**DOI:** 10.3390/s25010084

**Published:** 2024-12-26

**Authors:** Haodong Li, Zhengkai Yin, Changsheng Chen

**Affiliations:** 1 Shenzhen Research Institute of Northwestem Polytechnical University, Shenzhen 518057, China; yinzhengkai@mail.nwpu.edu.cn; 2 AVIC Aeronautics Computing Technology Research Institute, Xi’an 710069, China; ccsheng@avic.com; 3 Department of Communication Engineering, School of Electronic Information, Chang’an Campus, Northwestern Polytechnical University, Xi’an 710072, China

**Keywords:** unmanned aerial vehicle, non-orthogonal multiple access, edge computing, backscatter communication, delay minimization

## Abstract

The rapid deployment and enhanced communication capabilities of unmanned aerial vehicles (UAVs) have enabled numerous real-time sensing applications. These scenarios often necessitate task offloading and execution under stringent transmission delay constraints, particularly for time-critical applications such as disaster rescue and environmental monitoring. This paper investigates the improvement of MEC-based task offloading services in energy-constrained UAV networks using backscatter communication (BackCom) with non-orthogonal multiple access (BAC-NOMA). The proposed BAC-NOMA protocol allows uplink UAVs to utilize downlink signals for backscattering tasks instead of transmitting through uplink NOMA. A resource allocation problem is formulated, aimed at minimizing offloading delays for uplink users. By converting the initially non-convex problem into a convex one, an iterative algorithm is developed to solve it. Simulation results demonstrate that the proposed protocol significantly reduces offloading delays relative to existing benchmarks.

## 1. Introduction

Advancements in communication technologies and the increased availability of unmanned aerial vehicles (UAVs) have significantly increased the demand for real-time data processing across various applications [1,2,3]. UAVs are extensively employed in disaster management, environmental monitoring, and surveillance, where prompt data processing and decision-making are essential [4].

There is increasing interest in deploying UAVs for various time-sensitive applications due to their flexibility and rapid deployment capabilities. However, these applications often involve complex computations that require substantial processing power, which UAVs lack because of their limited onboard computational resources and battery life. Multiple-access edge computing (MEC) provides a solution by offloading tasks to nearby edge servers, thereby reducing the computational burden on UAVs and ensuring timely data processing. MEC has emerged as a crucial technology for the envisioned sixth-generation (6G) mobile network, where powerful servers are deployed at the edge of base stations (BS) [5,6]. This setup enables UAVs to offload their computationally intensive tasks to edge servers, which then process the data and return the results.

Substantial improvements in communication services are required to accommodate the large traffic volumes associated with MEC offloading. Recent studies have shown that combining non-orthogonal multiple access (NOMA) and MEC can significantly enhance spectrum efficiency and reduce latency [7,8,9]. NOMA is envisioned as a promising multiple-access (MA) technology for wireless communication systems which breaks the orthogonality of conventional MA techniques such as orthogonal frequency-division multiple access (OFDMA) and time-division multiple access (TDMA) [10,11]. In addition, it enables multiple users to share the same radio resource, improving the system’s overall achievable rate.

Despite the benefits of MEC, the energy consumption of UAVs remains a critical issue, as it directly impacts their operational time and overall performance. To address this challenge, backscatter communication (BackCom) has been introduced as a potential solution to reduce energy consumption in wireless communication [12,13,14]. By reflecting and modulating ambient RF signals, BackCom significantly decreases the energy required for data transmission. Therefore, combining BackCom with NOMA (BAC-NOMA) is considered a viable approach to enhance offloading services for resource-limited devices. This paper proposes integrating BackCom with NOMA (BAC-NOMA) to enhance MEC task offloading efficiency in UAV networks, aiming to achieve reduced transmission delays and improved energy efficiency.

### 1.1. Related Works

Previous studies have explored various BackCom protocols for enhancing uplink transmission scenarios. For instance, the authors of [15] addressed the challenge of maximizing computational efficiency (CE) in BackCom-aided MEC systems, introducing a novel user-cooperative offloading protocol that allows users to alternate between active transmission and BackCom modes across different time slots. In [16], the authors considered a BackCom-assisted MEC network in which edge users are equipped with both a backscatter circuit and an active transmission circuit to perform a hybrid BackCom and harvest-then-transmit (HTT) protocol. A weighted-sum computation bit maximization problem was formulated to jointly optimize the BackCom reflection coefficient, active transmission power, local computing frequency, and operation times for BackCom and active transmission.

Several studies have investigated the combination of NOMA with BackCom transmission. The authors of [13] proposed a BAC-NOMA scheme in which the NOMA users receive signals from both the BS and the BD excited by the downlink transmission. A NOMA-enabled backscatter communication protocol was proposed in [17] to maximize average energy efficiency. In this approach, user pairing, BackCom reflection coefficients, and the read’s transmit power are optimized using an alternating optimization technique. The full-duplex (FD) BAC-NOMA scheme was considered in [12], where downlink transmission was used to excite the circuit of BDs, enabling them to perform uplink transmission to the BS. The uplink sum rate maximization problem was studied, and the optimal solution was obtained by reformulating the problem as a linear programming problem.

Moreover, a number of studies have advanced schemes involving MEC-assisted BackCom-equipped UAV networks. In [18], a UAV was deployed as a data collector, and uplink NOMA was utilized to serve a large number of BackCom devices. By finding the optimal UAV flight height, their study maximized the number of decoded bits while minimizing the flight time. In [19], a sum rate maximization problem was established in which multiple BackCom devices are divided into clusters to share the same radio resource, allowing them to communicate with the UAV through NOMA transmission. The authors of [20] considered ma BackCom system based on the NOMA protocol and assisted by a full-duplex UAV. Their study maximized the sum rate by optimizing the reflection coefficient (RC) of BDs and the location of the UAV, and also proposed an iterative algorithm to solve the sum rate problem by using the block coordinated descent (BCD) technique and quadratic transform algorithm. Similarly, the authors of [21] considered the energy collection time, gateway amplification factor, and UAV position to establish a sum rate maximization resource allocation model.

Due to UAVs’ fast mobility and on-board processing capability, UAV-assisted wireless sensing networks can be reshaped dynamically to meet the time-varying traffic demands and quality provisioning requirements of ground users (GUs). This calls for joint optimization of UAV transmission control and trajectory planning in order to improve the freshness of information at the sink node (e.g., the BS). The authors of [22] considered multiple UAVs that assist in sensing data transmissions from the GUs to a remote base station. Each UAV collects sensing data from the GUs via low-power backscatter communications, and then forwards these data to the remote BS via NOMA transmissions. The long-term time-averaged age of information (AOI) can be minimized by jointly optimizing the GU access control, UAV beamforming, and trajectory planning strategies.

Several studies have investigated the resource optimization problem of backscatter communication network systems. The authors of [23] maximized the minimum EE of the system by optimizing the transmit power from the base station to the users along with the reflection coefficient. Their study proposed an algorithm for jointly allocation the power and backscatter reflection coefficient to maximize the EE. In [24], the authors proposed a backscatter communication–mobile edge computing (BC-MEC) network system based on a NOMA communication mode. To maximize the computation energy efficiency (CEE) of the system, their study optimized the backscatter coefficient of each mobile device along with the backscatter communication duration, direct offloading duration, MEC server processing time, local processing time, direct offloading power of each mobile device, calculation frequency of the MEC server, and local calculation frequency of each mobile device. In [25], the authors suggested the use of intelligent reflecting surface (IRS) and ambient backscatter communication (ABC) technologies, and proposed an IRS-assisted relay transmission scheme. In this approach, the energy efficiency is maximized by jointly optimizing the phase shifts of IRS reflecting elements and the power allocation of NOMA users. Furthermore, the authors of [26] maximized the EE of a UAV-assisted backscatter communication network by jointly optimizing time resource allocation and UAV trajectory, then adopting a Dinkelbach-based iterative algorithm to derive the optimal solution.

Despite the valuable insights these studies provide for the development of NOMA technology, they often overlook the importance of minimizing task offloading delay in UAV networks, especially in energy-constrained environments and those that demand real-time performance. The main difference between this paper and existing work is that we propose a novel NOMA-based drone network task offloading service improvement scheme that incorporates BackCom technology. Unlike previous studies focused on maximizing either energy efficiency or computational efficiency, we aim to significantly reduce transmission delay in energy-constrained UAV networks by providing MEC task offloading services. Furthermore, we consider the full-duplex (FD) interference scenario, where the environment signal is considered as both an interference source and an energy source, while also complying with downlink user QoS constraints.

### 1.2. Motivation and Contributions

Existing works have primarily focused on maximizing the energy efficiency (EE) [13], CE [15], or sum rate [12,27] of BAC-NOMA schemes. Furthermore, the proposal in [17] only allows two BackCom devices to share the same time-frequency resource block using NOMA. In this paper, we address the delay minimization problem in a hybrid BAC-NOMA-assisted UAV offloading scenario comprising one BS, one downlink user, and *K* UAVs.

In the proposed scenario, the UAVs are equipped with BackCom devices to backscatter signals; in particular, while the base station transmits to other users, the downlink transmission signal can excite the circuits of the BDs to generate energy for the UAVs, enabling task uploads to the MEC server. By leveraging the powerful computing capability of the MEC-equipped BS and BAC-NOMA, the UAVs can offload their detected raw data to the server for further processing with fairly low energy consumption. Due to FD interference, these BDs may not be able to complete the offloading operation when the downlink transmission is active; therefore, a second time duration is scheduled for offloading using NOMA uplink transmission. By applying the Dinkelbach method and quadratic transformation, an iterative algorithm is proposed to obtain the suboptimal solution to the original non-convex problem. A compared between benchmarks and simulation results demonstrates that the proposed protocol can significantly reduce offload latency.

The contributions of this paper are summarized as follows:

- We propose a novel hybrid offloading scheme that integrates backscatter communication (BackCom) with non-orthogonal multiple access (NOMA) with the aim of enhancing task offloading services in MEC-assisted UAV networks.
- We consider the FD interference scenario, in which the ambient signal is treated as both interference and as an energy-exciting source. Meanwhile, the achievable rate for the downlink user is guaranteed by a QoS constraint.
- We present the delay minimization problem within a hybrid BAC-NOMA framework, and consider both BackCom transmission and NOMA uplink transmission for optimization.
- We address the delay minimization problem in a hybrid BAC-NOMA framework. To handle the non-convex nature of the delay minimization problem, we employ the Dinkelbach method and quadratic transformation, reformulating the problem into a convex form for more efficient solving.
- We conduct simulations to evaluate the proposed hybrid BAC-NOMA scheme’s performance in comparison to the pure uplink NOMA scheme and pure BAC-NOMA scheme, demonstrating significant improvements in terms of lower average delay.

### 1.3. Organizations

The remainder of this paper is organized as follows: Section 2 introduces the system model; Section 3 presents the formulation of the proposed MEC delay minimization problem; Section 4 provides the details of the proposed UAV-based BAC-NOMA MEC offloading scheme and corresponding algorithms; Section 5 presents the simulation results and analysis; finally, Section 6 concludes the paper.

## 2. System Model

**Figure 1:**
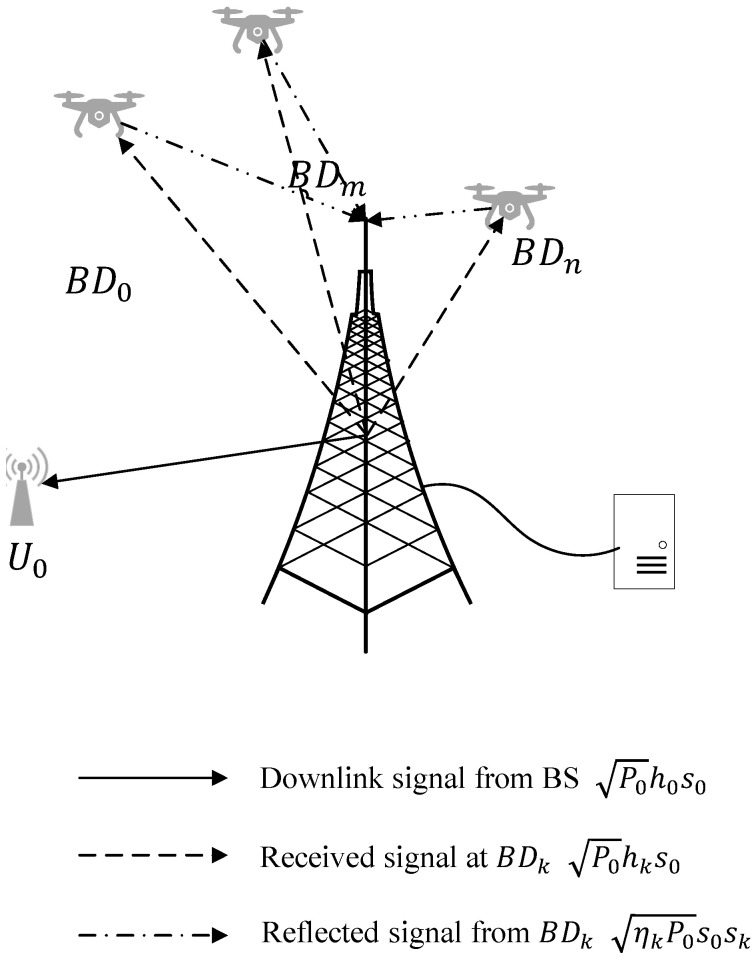
System model of BackCom transmission.

Consider a base station (BS) equipped with a multiple-access edge computing (MEC) server. The BS serves one downlink user $U_{0}$ along with *K* uplink BackCom-UAVs, denoted by ${\mathrm{BD}}_{k}$, where k∈{1,…,K}, as illustrated in Figure 1. During time slot $t_{0}$, the BS initiates downlink transmission to $U_{0}$. The downlink transmit power is denoted by $P_{0}$, while the received signal at ${\mathrm{BD}}_{k}$ can be expressed as $\sqrt{P_{0}}h_{k}s_{0}$. Here, $h_{k}$ represents the channel gain between the BS and ${\mathrm{BD}}_{k}$, with $s_{0}$ as the downlink signal to $U_{0}$ with unit power.

By utilizing the BackCom protocol, the downlink signal can activate the circuits of the BDs; the reflected signal from ${\mathrm{BD}}_{k}$ is provided by

(1) $$x_{BD_{k}}=\sqrt{\eta_{k}P_{0}}h_{k}s_{0}s_{k},$$

where $\eta_{k}\in \left[0,1\right]$ denotes the BackCom reflection coefficient and $s_{k}$ is the signal of $BD_{k}$ with unit power.

Therefore, the reflected signal received at the BS can be represented by [12]

(2) $$y_{BS}=\sum_{k=1}^{K}\sqrt{\eta_{k}P_{0}}h_{k}^{2}s_{0}s_{k}+I_{SI}+n_{BS},$$

where $I_{SI}$ denotes the full-duplex (FD) self-interference, which is assumed to follow a complex Gaussian distribution and to satisfy $I_{SI}∼\mathrm{CN}(0,\alpha P_{0}|h_{SI}{|}^{2})$, where α∈[0,1] represents the amount of residual interference. The complex additive white Gaussian noise (AWGN) at the receiver is denoted by $n_{BS}∼\mathrm{CN}\left(0,\sigma^{2}\right)$, and has zero mean and variance $\sigma^{2}$. Without loss of generality, the BDs are arranged in descending order based on their effective channel gains, satisfying

(3) $${\left|h_{1}\right|}^{2}\geq {\left|h_{2}\right|}^{2}\geq \ldots \geq {\left|h_{k}\right|}^{2}\geq \ldots \geq {\left|h_{K-1}\right|}^{2}\geq {\left|h_{K}\right|}^{2},\;\forall k\in \left\{1,\ldots ,K\right\}.$$

The successive interference cancellation (SIC) technique is employed for NOMA decoding. The BS prioritizes decoding the signals of those BDs with better channel gains, and treats the signals from BDs with worse channel gains as interference. Assuming that the downlink signal $s_{0}$ is perfectly known by the BS, the achievable rate of ${\mathrm{BD}}_{k}$ can be expressed as

(4) $$\begin{array}{cc} \; & r_{b,k}=B\log_{2}\left(1+\frac{\eta_{k}P_{0}|h_{k}{|}^{4}{\left|s_{0}\right|}^{2}}{\sum_{j=k+1}^{K}\eta_{j}P_{0}|h_{j}{|}^{4}|s_{0}{|}^{2}+\alpha P_{0}{\left|h_{SI}\right|}^{2}+\sigma^{2}}\right), \end{array}$$

where 0≤α≪1 denotes the amount of FD residual self-interference [12].

Moreover, because BackCom transmission characteristics introduce additional interference to the downlink user, the received signal at $U_{0}$ can be written as [12]

(5) $$y_{0}=\sqrt{P_{0}}h_{0}s_{0}+\sum_{k=1}^{K}\sqrt{\eta_{k}P_{0}}g_{k}h_{k}s_{0}s_{k}+n_{U_{0}},$$

where $h_{0}$ is the channel gain between the BS and $U_{0}$, $g_{k}$ represents the channel gain between $U_{0}$ and ${\mathrm{BD}}_{k}$, and $n_{U_{0}}∼\mathrm{CN}\left(0,\sigma^{2}\right)$ denotes the AWGN between $U_{0}$ and the BS. Thus, the achievable rate for $U_{0}$ is

(6) $$r_{0}=B\log_{2}\left(1+\frac{P_{0}{\left|h_{0}\right|}^{2}}{\sum_{k=1}^{K}\eta_{k}P_{0}|g_{k}{|}^{2}{\left|h_{k}\right|}^{2}+\sigma^{2}}\right).$$

**Figure 2:**
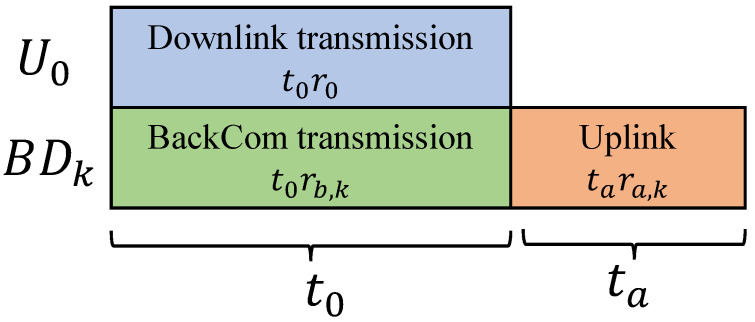
The proposed hybrid BackCom transmitting schedule.

In addition to BackCom transmission, this study considers an active uplink transmission scheme in which an extra time duration $t_{a}$ is allocated to the BDs for uplink transmission. The proposed transmission schedule is shown in Figure 2. It is assumed that offloading may not be completed within $t_{0}$, as completing offloading within $t_{0}$ might require higher downlink power $P_{0}$ or longer transmission time $t_{0}$, potentially degrading the transmission service provided to $U_{0}$. Thus, the BDs can instead utilize an additional time duration $t_{a}$ for NOMA uplink transmission.

The achievable active transmission rate for ${\mathrm{BD}}_{k}$ can be expressed as

(7) $$r_{a,k}=B\log_{2}\left(1+\frac{p_{a,k}{\left|h_{k}\right|}^{2}}{\sum_{j=k+1}^{K}p_{a,j}{\left|h_{j}\right|}^{2}+\sigma^{2}}\right),$$

where $p_{a,k}$ denotes the active transmission power of ${\mathrm{BD}}_{k}$.

## 3. Problem Formulation

The aim of this paper is to minimize the MEC offloading delay of *K* BDs. The offloading delay is defined as $T=t_{0}+t_{a}$, where $t_{0}$ is assumed to be fixed and predetermined by the BS, as it is primarily assigned to the downlink transmission for $U_{0}$. We consider the offload tasks of *K* BDs to the MEC server through NOMA simultaneously; the time consumption $t_{a}$ for offloading the remaining data can be calculated as

(8) $$t_{a}=\frac{L_{k}-t_{0}r_{b,k}}{r_{a,k}},k\in \left\{1,\ldots ,K\right\},$$

where $L_{k}$ is the offloading data amount of ${\mathrm{BD}}_{k}$. Hence, based on the expression of $t_{a}$, the delay minimization problem can be formulated as follows:

(9a) $$\begin{array}{cccc} \left(P1\right) & \min_{P_{0},p^{a},\eta } & \; & t_{0}+t_{a} \end{array}$$

(9b) $$\begin{array}{cccc} \; & s.t. & \; & r_{0}\geq \gamma_{0}, \end{array}$$

(9c) $$\begin{array}{cccc} \; & \; & & 0\leq t_{a}p_{a,k}\leq E_{k,\max},\;\forall k \end{array}$$

(9d) $$\begin{array}{cccc} \; & \; & & 0\leq p_{a,k}\leq P_{a,\max},\forall k \end{array}$$

(9e) $$\begin{array}{cccc} \; & \; & & 0\leq P_{0}\leq P_{0,\max}, \end{array}$$

(9f) $$\begin{array}{cccc} \; & \; & & 0\leq \eta_{k}\leq 1,\;\forall k \end{array}$$

where $p^{a}≜\left[p_{a,1},p_{a,2},\ldots ,p_{a,k}\right]$ and $\eta ≜[\eta_{1},\eta_{2},\ldots ,\eta_{K}]$. The minimum achievable rate for the downlink transmission is guaranteed by (9b). Constraint (9c) ensures that the the energy consumption for offloading is limited by the energy of each BD. The power of uplink transmission for ${\mathrm{BD}}_{k}$ is limited in (9d). Constraint (9e) guarantees the range of the downlink transmission power, while (9f) limits the range of the reflection coefficient. Because (P1) is non-convex due to the fractional form of the objective function and coupled variables, a convex equivalent of this problem is developed to tackle the resource allocation problem in the next section.

## 4. Problem Transformation and Solution

In this section, we propose two approaches for transforming the original non-convex problem into two equivalent convex forms in order to adapt the two different offloading scenarios, i.e., pure BAC-NOMA offloading and hybrid BAC-NOMA offloading. If BDs are able to finish offloading within $t_{0}$, then the pure BAC-NOMA scheme becomes feasible and the second time duration $t_{a}=0$; otherwise, the hybrid BAC-NOMA scheme is adopted. Based on the above two schemes, an iterative based algorithm is proposed to efficiently obtain the resource allocation.

**Lemma** **1.** *We define $\tilde{L}=\sum_{k=1}^{K}L_{k}$, $t_{a}$, which is equivalent to the following expression:*

(10) $$t_{a}=\frac{\tilde{L}-t_{0}R_{b}}{R_{a}}$$

*where $R^{b}=\sum_{k=1}^{K}r_{b,k}$ and $R^{a}=\sum_{k=1}^{K}r_{a,k}$.*

**Proof.**  First, the remaining data after the first time duration $t_{0}$ are denoted as $\beta_{a,k}=L_{k}-t_{0}r_{b,k}$. As all BDs finish offloading their tasks simultaneously within the same transmission time $t_{a}$, the following relation holds:

(11) $$t_{a}=\frac{\beta_{a,1}}{r_{a,1}}=\frac{\beta_{a,2}}{r_{a,2}}=\ldots =\frac{\beta_{s,K}}{r_{a,K}}.$$

Because $\frac{\beta_{a,1}}{r_{a,1}}=\frac{\beta_{a,k}}{r_{a,k}}$, we have $\epsilon_{k}≜\frac{\beta_{a,k}}{\beta_{a,1}}=\frac{r_{a,k}}{r_{a,1}}$. Then, the following fraction can be constructed by multiplying the same term with both the numerator and denominator:

(12) $$\frac{\beta_{a,1}}{r_{a,1}}=\frac{\beta_{a,1}\left(1+\epsilon_{2}+\epsilon_{3}+\ldots +\epsilon_{K}\right)}{r_{a,1}\left(1+\epsilon_{2}+\epsilon_{3}+\ldots +\epsilon_{K}\right)}.$$

Therefore, the above equation can be written as

(13) $$\begin{array}{cc} \frac{\beta_{a,1}}{r_{a,1}}= & \frac{\beta_{a,1}\left(1+\frac{\beta_{a,2}}{\beta_{a,1}}+\ldots +\frac{\beta_{a,K}}{\beta_{a,1}}\right)}{r_{a,1}\left(1+\frac{r_{a,2}}{r_{a,1}}+\ldots +\frac{r_{a,K}}{r_{a,1}}\right)}=\frac{\sum_{k=1}^{K}\beta_{a,k}}{\sum_{k=1}^{K}r_{a,K}}, \end{array}$$

and the transmission time $t_{a}$ can be written as

(14) $$\begin{array}{cc} t_{a}= & \frac{\sum_{k=1}^{K}\left(L_{k}-t_{0}r_{b,k}\right)}{\sum_{k=1}^{K}r_{a,k}}\\ = & \frac{\sum_{k=1}^{K}L_{k}-t_{0}B\log_{2}\left(1+\frac{\sum_{k=1}^{K}\eta_{k}P_{0}|h_{k}{|}^{4}{\left|s_{0}\right|}^{2}}{\alpha P_{0}{\left|h_{SI}\right|}^{2}+\sigma^{2}}\right)}{B\log_{2}\left(1+\frac{\sum_{k=1}^{K}p_{a,k}{\left|h_{k}\right|}^{2}}{\sigma^{2}}\right)}. \end{array}$$

Based on (4), we can find the sum rate of ${\mathrm{BD}}_{K}$ and ${\mathrm{BD}}_{K-1}$ as follows:

(15) $$\begin{array}{cc} r_{b,K}+r_{b,K-1} & =B\log_{2}\left(1+\frac{\eta_{K}P_{0}|h_{K}{|}^{4}{\left|s_{0}\right|}^{2}}{\alpha P_{0}{\left|h_{SI}\right|}^{2}+\sigma^{2}}\right)\\ \; & +B\log_{2}\left(1+\frac{\eta_{K-1}P_{0}|h_{K-1}{|}^{4}{\left|s_{0}\right|}^{2}}{\eta_{K}P_{0}|h_{K}{|}^{4}|s_{0}{|}^{2}+\alpha P_{0}{\left|h_{SI}\right|}^{2}+\sigma^{2}}\right)\\ \; & =B\log_{2}\left(1+\frac{\eta_{K-1}P_{0}|h_{K-1}{|}^{4}|s_{0}{|}^{2}+\eta_{K}P_{0}|h_{K}{|}^{4}{\left|s_{0}\right|}^{2}}{\alpha P_{0}{\left|h_{SI}\right|}^{2}+\sigma^{2}}\right). \end{array}$$

Similarly, we have the sum rate

(16) $$\begin{array}{cc} r_{b,K-2}+ & r_{b,K-1}+r_{b,K}=B\log_{2}\left(1+\frac{\sum_{i=K-2}^{K}\eta_{i}P_{0}|h_{i}{|}^{4}{\left|s_{0}\right|}^{2}}{\alpha P_{0}{\left|h_{SI}\right|}^{2}+\sigma^{2}}\right). \end{array}$$

By repeating the above manipulations, the sum rate of *K* BDs using the backscatter transmission can be written as

(17) $$R_{b}=\sum_{k=1}^{K}r_{b,k}=B\log_{2}\left(1+\frac{\sum_{k=1}^{K}\eta_{k}P_{0}|h_{k}{|}^{4}{\left|s_{0}\right|}^{2}}{\alpha P_{0}{\left|h_{SI}\right|}^{2}+\sigma^{2}}\right).$$

Similar to (17), the sum rate for the active uplink transmission can be written as

(18) $$R_{a}=\sum_{k=1}^{K}r_{a,k}=B\log_{2}\left(1+\frac{\sum_{k=1}^{K}p_{a,k}{\left|h_{k}\right|}^{2}}{\sigma^{2}}\right).$$

By substituting (17) and (18) into (14), the offloading latency $t_{a}$ can be written as

(19) $$t_{a}=\frac{\tilde{L}-t_{0}R_{b}}{R_{a}}.$$

   □

### 4.1. Pure BAC-NOMA Case

If all BDs can finish offloading within $t_{0}$, and assuming that the backscatter transmission satisfies the constraint $L_{k}=t_{0}r_{b,k}$, then the second time duration $t_{a}$ will be zero. According to Lemma 1, the above constraint is equivalent to $\tilde{L}=t_{0}R_{b}$. Hence, the original problem becomes a feasibility problem, which can be formulated as

$$\begin{array}{cccc} \; & (P2)\; & \; & \mathrm{find}\;P_{0},p_{r,k} \end{array}$$

(20a) $$\begin{array}{cccc} \; & s.t. & \; & \sum_{k=1}^{K}p_{r,k}|h_{k}{|}^{4}{\left|s_{0}\right|}^{2}-\gamma \left(\alpha P_{0}{\left|h_{SI}\right|}^{2}+\sigma^{2}\right)=0, \end{array}$$

(20b) $$\begin{array}{cccc} \; & \; & & P_{0}|h_{0}{|}^{2}-\tilde{\gamma_{0}}\sum_{k=1}^{K}p_{r,k}|g_{k}{|}^{2}{\left|h_{k}\right|}^{2}-\tilde{\gamma_{0}}\sigma^{2}\geq 0, \end{array}$$

(20c) $$\begin{array}{cccc} \; & \; & & 0\leq P_{0}\leq P_{0,\max}, \end{array}$$

(20d) $$\begin{array}{cccc} \; & \; & & 0\leq p_{r,k}\leq P_{0},\;\forall k, \end{array}$$

where $p_{r,k}=\eta_{k}P_{0}$, $\gamma =2^{\frac{\tilde{L}}{t_{0}B}-1}$, and $\tilde{\gamma_{0}}=2^{\frac{\gamma_{0}}{B}}-1$. It is evident that the above problem is convex, meaning that the feasibility can be found using an optimization solver.

### 4.2. Hybrid BAC-NOMA Case

When $\tilde{L}\geq t_{0}R_{b}$, the BDs cannot finish offloading during $t_{0}$, and the remaining data must be offloaded during $t_{a}$. Because the objective function in (P1) is fractional and non-convex, the Dinkelbach method [28] is leveraged to transform the original problem into an equivalent objective function. The principle of the Dinkelbach’s method is to solve a non-convex delay minimization problem in which the objective function involves a ratio of two variables in the term $\frac{\tilde{L}-t_{0}R_{b}}{R_{a}}$. The Dinkelbach method helps to convert the fractional problem into a more tractable form, making it easier to apply standard optimization techniques to find the optimal solution. The fractional optimization problem can be transformed into a non-fractional problem by introducing a new auxiliary variable. Starting with an initial value for the auxiliary variable, we then iteratively solve the transformed non-fractional problem. After solving the non-fractional problem, we update the value of the auxiliary variable based on the result. The updated auxiliary variable is used in the next iteration. The iterations continue until the auxiliary variable converges to the optimal ratio.

For instance, the fractional-format non-convex problem is transformed into the following optimization problem:

(21a) $$\begin{array}{cccc} \left(P3\right) & \min_{P_{0},p^{a},\eta } & \; & \begin{array}{cc} \tilde{L} & -t_{0}R_{b}-\mu R_{a} \end{array} \end{array}$$

(21b) $$\begin{array}{cccc} \; & s.t. & \; & 0\leq \mu p_{a,k}\leq E_{k,\max} \end{array}$$

(21c) $$\begin{array}{cccc} \; & \; & & r_{0}\geq \gamma_{0}, \end{array}$$

(21d) $$\begin{array}{cccc} \; & \; & & 0\leq p_{a,k}\leq P_{a,\max},\forall k \end{array}$$

(21e) $$\begin{array}{cccc} \; & \; & & 0\leq P_{0}\leq P_{0,\max}, \end{array}$$

(21f) $$\begin{array}{cccc} \; & \; & & 0\leq p_{r,k}\leq P_{0},\;\forall k \end{array}$$

where μ is an auxiliary variable determined by the proposed iterative algorithm presented below.

Problem (P3) is still non-convex, due to the existence of FD interference in the second term in $R_{b}$; as the goal is to minimize $\tilde{L}-t_{0}R_{b}-\mu R_{a}$, and as $R_{b}$ is the only non-convex term in this objective function, it is equivalent to finding the maximum $R_{b}$ to minimize the objective function. Hence, to further simplify the problem, the quadratic transform [29] is applied to the objective function of (P3). Quadratic transformation is a technique used to convert non-convex optimization problems into convex problems by rewriting certain terms as quadratic functions. In this paper, $R_{b}$ can be rewritten in quadratic form to solve the equivalent problem.

Thus, finding the maximum for $R_{b}$ is equivalent to transferring $R_{b}$ into the following quadratic expression:

(22) $$q\left(y\right)=t_{0}B\log_{2}\left\{\begin{array}{cc} 1+2y & \sqrt{\sum_{k=1}^{K}p_{r,k}|h_{k}{|}^{4}{\left|s_{0}\right|}^{2}}-y^{2}\left(\alpha P_{0}{\left|h_{SI}\right|}^{2}+\sigma^{2}\right) \end{array}\right\}$$

where *y* is an auxiliary variable which is updated iteratively. For given $p_{r,k}$ and $P_{0}$, the optimal $y^{*}$ to maximize $q(y^{*})$ can be determined based on the principle of quadratic functions using

(23) $$y^{*}=\frac{\sqrt{\sum_{k=1}^{K}p_{r,k}|h_{k}{|}^{4}{\left|s_{0}\right|}^{2}}}{\alpha P_{0}{\left|h_{SI}\right|}^{2}+\sigma^{2}}.$$

The reformulated problem can be expressed as follows:

(24a) $$\begin{array}{cccc} \left(P4\right) & \min_{\begin{array}{c} P_{0},p^{a},p^{r} \end{array}}\; & \; & \begin{array}{cc} \;F(\mu ) & =\tilde{L}-\mu B\log_{2}\left(1+\frac{\sum_{k=1}^{K}p_{a,k}{\left|h_{k}\right|}^{2}}{\sigma^{2}}\right)\\ \; & -t_{0}B\log_{2}\left\{\begin{array}{cc} 1+2y & \sqrt{\sum_{k=1}^{K}p_{r,k}|h_{k}{|}^{4}{\left|s_{0}\right|}^{2}}-y^{2}\left(\alpha P_{0}{\left|h_{SI}\right|}^{2}+\sigma^{2}\right) \end{array}\right\} \end{array} \end{array}$$

(24b) $$\begin{array}{cccc} \; & s.t. & \; & 0\leq p_{a,k}\leq P_{a,\max},\forall k \end{array}$$

(24c) $$\begin{array}{cccc} \; & \; & & 0\leq P_{0}\leq P_{0,\max}, \end{array}$$

(24d) $$\begin{array}{cccc} \; & \; & & P_{0}|h_{0}{|}^{2}-\tilde{\gamma_{0}}\sum_{k=1}^{K}p_{r,k}|g_{k}{|}^{2}{\left|h_{k}\right|}^{2}-\tilde{\gamma_{0}}\sigma^{2}\geq 0, \end{array}$$

(24e) $$\begin{array}{cccc} \; & \; & & 0\leq p_{r,k}\leq P_{0},\;\forall k, \end{array}$$

(24f) $$\begin{array}{cccc} \; & \; & & 0\leq \mu p_{a,k}\leq E_{k,\max}. \end{array}$$

Hence, for fixed auxiliary variables *y* and μ, problem (P4) becomes a convex problem. CVX can then be utilized to efficiently solve this problem [30,31]. By providing a feasible initial $y^{\left(0\right)}$, the optimal $P_{0}^{*}$, $p_{r,k}^{*}$, and $p_{a,k}^{*}$ can be obtained based on the given $y^{\left(0\right)}$. Then, *y* can be updated for next iteration based on (23) with the obtained optimization variables.

Therefore, an algorithm is proposed to iteratively update the power allocation solution and the iterative variable μ. The proposed algorithm begins by initializing key parameters, including *l*, μ, $P_{0}$, and $p_{r,k}$, then checking whether the pure BAC-NOMA scheme is feasible. This involves determining whether all UAVs can complete their offloading within the initial time slot $t_{0}$. If feasible, the algorithm proceeds with pure backscatter transmission while solving the corresponding optimization problem to obtain the power allocation for downlink and BackCom transmissions. If the pure BAC-NOMA scheme is not feasible, it initializes the downlink power $P_{0}$ and reflection power $p_{r,k}$ for each UAV with feasible values, iteratively updates the auxiliary variable μ and *y*, and solves the optimization problem F(μ) to find the optimal power allocations.

The procedure of the proposed scheme is summarized in Algorithm 1. The feasibility of (P2) is verified first in order to determine whether all users can finish offloading via BAC-NOMA within time $t_{0}$. With the increment of *l*, the Dinkelbach iterative variable μ finally converges to the ε-optimal $\mu^{*}$, where the ε-optimal $\mu^{*}$ can be achieved if $F(\mu^{*})\leq \varepsilon$. The convergence of the Dinkelbach based algorithm was proved in [28], and our simulation results presented in the next section show that the proposed algorithm is able to converge within a few iterations.
**Algorithm 1** Iterative resource allocation algorithm  1:Input: $h_{k}$, $g_{k}$, α, $L_{k}$, $E_{k,\max}$, $\gamma_{0}$, $t_{0}$, ε  2:Set l=0, μ=+∞  3:**if** (P2) is feasible **then**  4:    Pure backscatter transmission is adopted.  5:    Solve (P2) and obtain $P_{0}^{*}$ and $p_{r,k}^{*}$.  6:**else**   7:   Initialize $P_{0}$, $p_{r,k}$ with feasible values.   8:   **repeat**  9:       l=l+110:       Update $y^{\left(l\right)}$ by (23).11:       Solve problem (P4) with fixed μ, and obtain $P_{0}^{\left(l\right)}$, $p_{r,k}^{\left(l\right)}$, and $p_{a,k}^{\left(l\right)}$.12:       Solve $F(\mu^{\left(l\right)})$.13:       updata μ as $\mu^{\left(l\right)}=\frac{\tilde{L}-t_{0}B\log_{2}\left(1+\frac{\sum_{k=1}^{K}p_{r,k}^{\left(l\right)}|h_{k}{|}^{4}{\left|s_{0}\right|}^{2}}{\alpha P_{0}^{\left(l\right)}{\left|h_{SI}\right|}^{2}+\sigma^{2}}\right)}{B\log_{2}\left(1+\frac{\sum_{k=1}^{K}p_{a,k}^{\left(l\right)}{\left|h_{k}\right|}^{2}}{\sigma^{2}}\right)}$.14:    **until** $F(\mu^{\left(l\right)})\leq \varepsilon$.15:    $P_{0}^{*}=P_{0}^{\left(l\right)}$, $p_{r,k}^{*}=p_{r,k}^{\left(l\right)}$, and $p_{a,k}^{*}=p_{a,k}^{\left(l\right)}$.16:**end if**

The time complexity of this algorithm scales with respect to the number of UAVs, the number of iterations required for convergence in the Dinkelbach method, and the number of iterations in the quadratic transformation step. The step to initialize the parameters involves a constant number of operations, meaning that it contributes O(1) to the overall time complexity. The feasibility check involves determining whether the pure BAC-NOMA scheme will be sufficient; if there are *K* UAVs, the complexity of this step is O(K), as we need to solve problem (P2). The number of iterations required for convergence in the quadratic transformation step is generally dependent on the size of the problem, typically requiring a time per O(K) iteration. Because there might be $I_{quad}$ iterations, the time complexity for this step is $O(I_{quad}\cdot K)$. The number of iterations in the Dinkelbach method depends on how quickly the objective function converges to the optimal solution. The total time complexity for the Dinkelbach method is $O(I_{Dink}\cdot K)$, where $I_{Dink}$ is the number of iterations required for convergence. To summarize, the total time complexity of Algorithm 1 is dominated by the time complexity of the iterative steps, and can be expressed as

(25) $$O(I_{quad}\cdot K+I_{Dink}\cdot K+K+1),$$

which means that the time complexity scales linearly with the number of UAVs.

## 5. Simulation Results

In this section, simulation results are presented and discussed to evaluate the proposed hybrid NOMA-BackCom scheme. The simulation settings are listed as follows.

The communication channels between the BS, UAVs (denoted as ${\mathrm{BD}}_{k}$), and downlink user $U_{0}$ are modeled using Rayleigh fading for all link types. The channel gain for each link is calculated using a distance-based path loss model

(26) $$h_{k}={\tilde{h}}_{k}d_{k}^{-\frac{\chi }{2}},$$

where ${\tilde{h}}_{k}$ is the Rayleigh fading coefficient, $d_{k}$ is the distance between the BS and ${\mathrm{BD}}_{k}$, and χ is the path loss exponent, which was set to 3.76 for all simulations. Similarly, the channel gains $h_{0}$ (BS to $U_{0}$) and $g_{k}$ (UAV to $U_{0}$) are calculated using the same model with the respective distances, in which the distance between $U_{0}$ and ${\mathrm{BD}}_{k}$ is denoted by ${\tilde{d}}_{k}$. The channel conditions, including fading coefficients, path loss, and distances, were randomly generated for each simulation run to simulate a realistic wireless communication environment. The maximum uplink transmission power and energy constraints were applied to each UAV, then the offloading task was simulated based on these parameters.

The transmission radius of the BS is 50 m, and all BDs as well as $U_{0}$ are located within the disc region. The AWGN power is $\sigma^{2}=BN_{0}$, where B=5 MHz, and $N_{0}=-94$ dBm/Hz denotes the AWGN spectrum density. The error tolerance was set to $\varepsilon =10^{-4}$, and the simulation results were obtained after $10^{3}$ realizations. The summarized parameters are listed in Table 1.

**Figure 3:**
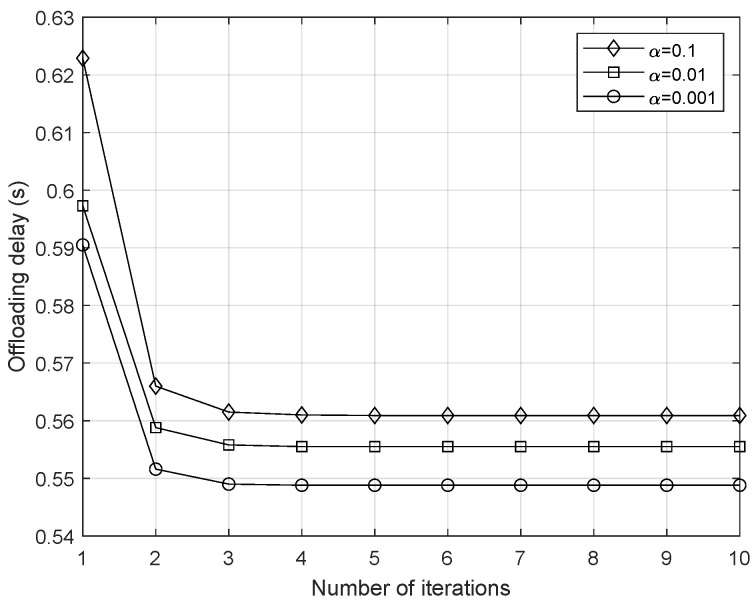
Comparison of convergence performance and delay.

The convergence performance of the proposed Algorithm 1 and the effect of the amount of FD residual self-interference are demonstrated in Figure 3. In this figure, the data length of each user $L_{k}$ is set to $10^{6}$ bits, the initial time duration $t_{0}=0.5$ s, and the maximum uplink transmission power is $p_{a,\max}=0.5$ watts. According to the simulation results, the algorithm converges efficiently within a few iterations. Additionally, the amount of FD residual self-interference impacts the offloading delay, as higher residuals can decrease the achievable rate of each BD, thereby increasing the remaining data amount for offloading in the second duration.

**Figure 4:**
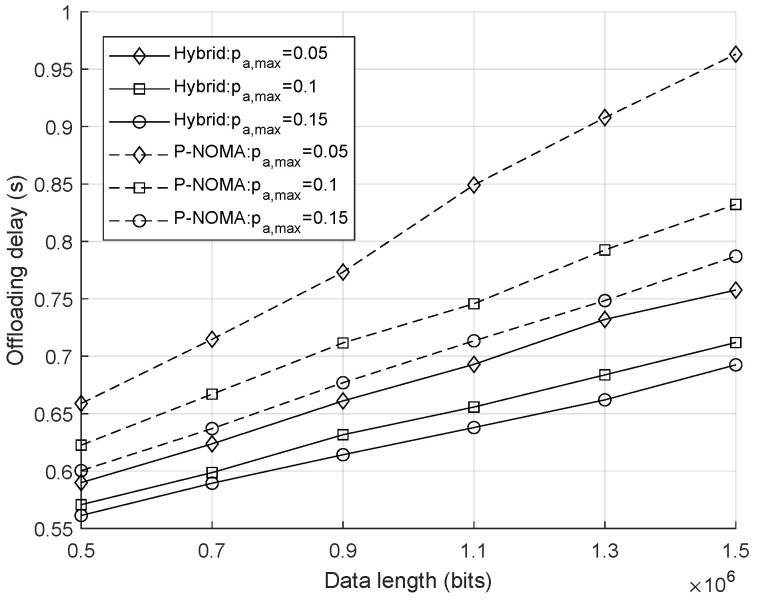
Average offloading delay achieved by hybrid offloading and pure NOMA uplink transmission as a function of offloading data length.

As shown in Figure 4, the relationship between the average offloading delay and the data length of each user is analyzed under the fixed QoS constraint $\gamma_{0}=2$ Mbit/s and $E_{\max}=0.1$ joules. The pure NOMA scheme is considered as a benchmark, where BDs do not perform BackCom during $t_{0}$, implying that $\eta_{k}=0,\;\forall k$. For both transmission schemes, the average delay increases with the growth of offloading data length. The dashed lines, representing pure NOMA uplink transmission, show a higher average offloading delay compared to the solid line representing the hybrid offloading scheme. The proposed method consistently achieves the lowest average offloading delay across varying data lengths. This efficiency is attributed to the optimal combination of backscatter communication and NOMA, which allows UAVs to utilize downlink signals for backscattering tasks. Our hybrid scheme effectively reduces the time required for uplink transmission by leveraging existing downlink resources and active uplink transmission, leading to a more efficient offloading process. The average delay for the pure NOMA scheme is significantly higher, particularly as the data length increases. This increase is primarily due to the lack of backscatter capabilities, which limits the available resources for offloading. As a result, UAVs relying solely on uplink NOMA must consume more time for uplink transmission, leading to longer delays. Moreover, a higher power budget for offloading can significantly reduce the system delay in both cases.

**Figure 5:**
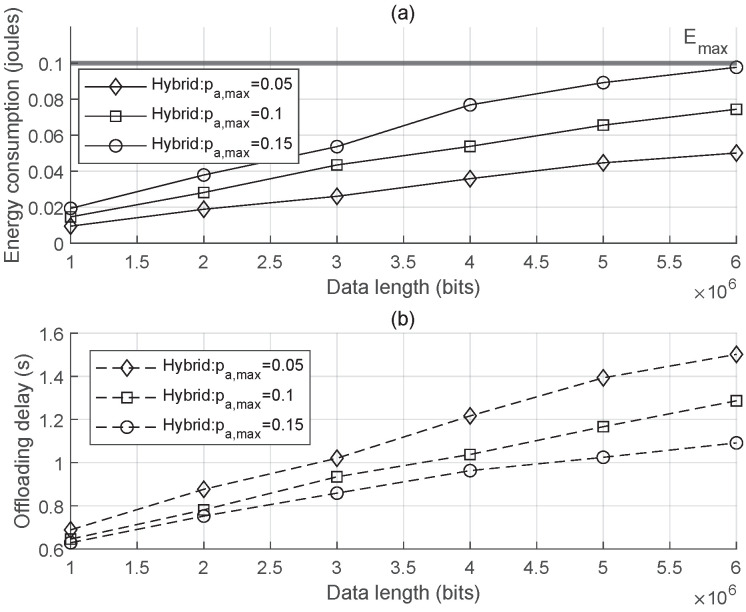
Average offloading energy consumption (**a**) and average offloading delay (**b**) as functions of offloading data length.

In Figure 5, the maximum energy budget for each user is set to $E_{k,max}=0.1$ joules. Figure 5a illustrates the relationship between the offloading data length and the energy consumption. As more data are offloaded to the BS, each device consumes more energy for uplink transmission, eventually reaching a point where the energy is fully utilized for each device and task offloading becomes infeasible due to the constrained energy if more than that amount of data need to be uploaded. When comparing the curve with $p_{a,\max}=0.05$ w, curves with higher uplink transmit power budget $p_{a,\max}$ demonstrate increased energy consumption for offloading the same amount of data. This is because the objective function focuses on minimizing the offloading delay, meaning that each user tends to utilize higher transmit power, as shown in Figure 5b. The energy consumption increases monotonically with the transmit power $p_{a,k}$, which verifies the proposition.

## 6. Conclusions

In this study, we address the problem of UAV offloading delay minimization in hybrid BAC-NOMA-assisted MEC networks. To overcome the non-convex nature of the initial problem, we utilize the Dinkelbach method and quadratic transformation to reformulate it into a convex problem. Our simulation results demonstrate the effectiveness of BackCom transmission and the performance advantages of the proposed hybrid scheme over the pure uplink NOMA scheme, particularly in reducing average delay. Our results suggest that the hybrid scheme significantly surpasses the pure NOMA approach, providing a more efficient solution for delay-sensitive applications in MEC-based UAV networks. This study sets the foundation for future research aimed at further enhancing the efficiency and effectiveness of hybrid offloading schemes in UAV communication networks.

## Figures and Tables

**Table 1:** Simulation parameters.

Parameter	Value
Channel Model	Rayleigh fading with path-loss exponent of 3.76
Transmission Radius	50 m
AWGN Power	$\sigma^{2}=BN_{0}$ with B=5 MHz, $N_{0}=-94$ dBm/Hz
Maximum Energy Budget per UAV	$E_{k,\max}=0.1$ joules
Data Length for each UAV	$L_{k}$ bits
Number of UAVs	*K*
Downlink Power Limit	$P_{0,\max}=1$ watt
Uplink Power Limit per UAV	$P_{a,\max}=0.5$ watts
Reflection Coefficient Range	$0\leq \eta_{k}\leq 1$
Quality of Service (QoS) Requirement	$\gamma_{0}$
Simulation Runs	$10^{3}$
Tolerance for Convergence	$\epsilon =10^{-4}$

## Data Availability

All data can be found within the article.

## Abbreviations

The following abbreviations are used in this manuscript:

UAV	Unmanned Aerial Vehicle
MEC	Multiple-Access Edge Computing
NOMA	Non-Orthogonal Multiple Access
BackCom	Backscatter Communication
HTT	Harvest-Then-Transmit
BAC-NOMA	Backscatter Communication with Non-Orthogonal Multiple Access
BS	Base Station
FD	Full-Duplex
RC	Reflection Coefficient
BCD	Block Coordinated Descent
GU	Ground Users
BC-MEC	Backscatter Communication–Mobile Edge Computing
CEE	Computation Energy Efficiency
IRS	Intelligent Reflecting Surface
ABC	Ambient Backscatter Communication
SI	Self-Interference
SIC	Successive Interference Cancellation
AWGN	Additive White Gaussian Noise
QoS	Quality of Service
EE	Energy Efficiency
